# The Effect of Halloysite Addition on the Material Properties of Chitosan–Halloysite Hydrogel Composites

**DOI:** 10.3390/gels5030040

**Published:** 2019-08-14

**Authors:** Yangyang Luo, David K. Mills

**Affiliations:** 1 Molecular Sciences & Nanotechnology, Louisiana Tech University, Ruston, LA 71272, USA; 2 School of Biological Sciences and the Center for Biomedical Engineering, Louisiana Tech University, Ruston, LA 71272, USA

**Keywords:** chitosan, composite, drug delivery, HNTs, hydrogel, gentamicin, nanocomposites, sustained release

## Abstract

Chitosan-based hydrogels are being widely used in biomedical applications due to their eco-friendly, biodegradable, and biocompatible properties, and their ability to mimic the extracellular matrix of many tissues. However, the application of chitosan hydrogels has been limited due to their inherent mechanical weakness. Halloysite nanotubes (HNTs) are naturally occurring aluminosilicate clay minerals and are widely used as a bulk filler to improve the performance characteristics of many polymeric materials. HNTs have also been shown to be a viable nanocontainer able to provide the sustained release of antibiotics, chemicals, and growth factors. This study’s objective was to develop a stable drug delivery chitosan/HNT nanocomposite hydrogel that is biocompatible, biodegradable, and provides sustained drug release. In this study, chitosan/HNTs hydrogels containing undoped or gentamicin-doped HNTs were combined in different wt./wt. ratios and cross-linked with tripolyphosphate. The effects of chitosan and HNTs concentration and combination ratios on the hydrogel surface morphology, degradability, and mechanical properties, as well as its drug release capability, were analyzed. The results clearly showed that the addition of HNTs improved chitosan mechanical properties, but only within a narrow range. The nanocomposite hydrogels provided a sustained pattern of drug release and inhibited bacterial growth, and the live/dead assay showed excellent cytocompatibility.

## 1. Introduction

Oral ingestion and intravascular injection of antibiotics have a lengthy application history and are primarily used in the control of infection post-surgical infection. However, there is a high risk of negative side effects [1,2]. These side effects are principally due systemic administration through the blood vascular system and not directly to the target tissue [3]. In many cases, frequent administration of antibiotics is required to achieve the dosage levels needed to eliminate the infection, and this regimen has the potential to severely impact unaffected tissues resulting in additional medical issues for the patient, such as gastric, hematological, neurological, dermatological, allergic and other disorders [3]. An implantable drug delivery system that can provide a defined drug load directly to the affected tissue is one strategy to resolve this problem. Key design considerations in building such a drug delivery system include biocompatibility, biodegradability, mechanical stability, and the ability to provide sustained drug release. In this study, chitosan and halloysite were used to construct and test composite hydrogels that differed in percent concentration of these materials.

Chitosan is a naturally derived hydrogel, usually produced by alkaline deacetylation and is biodegraded by human enzymes [4]. Chitosan has been proven to be non-toxic, possesses a lack of immunogenicity, possesses the ability to sequester bioactive factors, and has the capability of assembling a tissue-specific extracellular matrix (ECM) [4,5,6]. It also exhibits some antibacterial properties [7]. This antimicrobial ability makes chitosan a suitable candidate for implant coatings, wound dressing, and drug delivery applications, but chitosan has a major flaw which is its inherent mechanical weakness [8,9,10]. Many approaches, such as the addition of various polymers, [11,12] carbon nanotubes, [13,14] or clay nanoparticles [15,16], have been studied as a means to improve chitosan’s mechanical properties, and these additives increased the roughness of the scaffold which enhaced cell attachment, proliferation and differentiation [17,18,19].

Halloysite nanotubes (HNTs) are naturally occurring nanotubes composed of silica and alumina, [20] and exhibits a high degree of cytocompatibility hemocompatibility, and biocompatibility [21,22,23]. They are 1D nanomaterials with a unique hollow tubular morphology which has an external diameter of 50-200 nm, lumenal diameter of 5-30 nm and a length of 0.5-2 μm [20]. The electrokinetic behavior of HNT at pH 7 is defined by the negative surface potential of SiO_2_, with a small contribution from the positively-charged Al_2_O_3_ inner surface [24,25,26]. As a polymer filler, HNTs have been shown to significantly improve the material properties of polymers and resins such as alginate, [19] calcium phosphate cement, [27] epoxy, [28] nylon, [29] poly(methyl methacrylate), [30] and rubber [31]. The unique hollow tubular structure enables HNTs to be used as drug carriers. The HNT lumen can serve as a reservoir for the loading and release of a diverse set of biologically active molecules, including small molecules, enzymes, nucleic acids, and proteins [32,33,34,35,36]. Moreover, the loading capacity of HNTs can be further enlarged by chemical etching, thus increasing its cargo-carrying capacity [37].

Chitosan (CS) combined with different types of nanoparticles have been extensively studied [38]. Recent studies have shown that these nanocomposites are biocompatible, antimicrobial, and mucoadhesive and can be fabricated into various forms including coatings, [39] films, [40] hydrogels, [19] and membranes [41]. Furthermore, CS, with the addition of HNTs, has also been shown to significantly increase strength, tensile modulus, hardness, and toughness [17,42]. However, these studies only reported on the effects that HNT addition had on polymer mechanical properties, however, the influence of chitosan and HNT concentration and the corresponding impact of different percent combination of these materials on the mechanical properties and cellular behaviors has yet to be established.

In this study, chitosan was chosen to be cell growth scaffold due to its polycationic property and antibiotic potential [7,10]. The drug-carrying capacity of HNTs was employed as additives to improve chitosan hydrogel mechanical properties. The resultant changes in hydrogel surface structure, tensile strength, stiffness, and degradability were studied. Gentamicin was selected as a model drug to assess drug release in CS/HNTs hydrogels of different compositions. *Escherichia coli* (*E. coli*) and *Staphylococcus aureus* (*S. aureus*) were used as a means for testing the bacterial growth inhibition capacity of the different hydrogels and in estimating drug efficacy. Pre-osteoblasts (MC3T3) were selected to study the potential cytotoxicity of CS/HNTs nanocomposites.

## 2. Results

### 2.1. SEM

**Figure 1:**
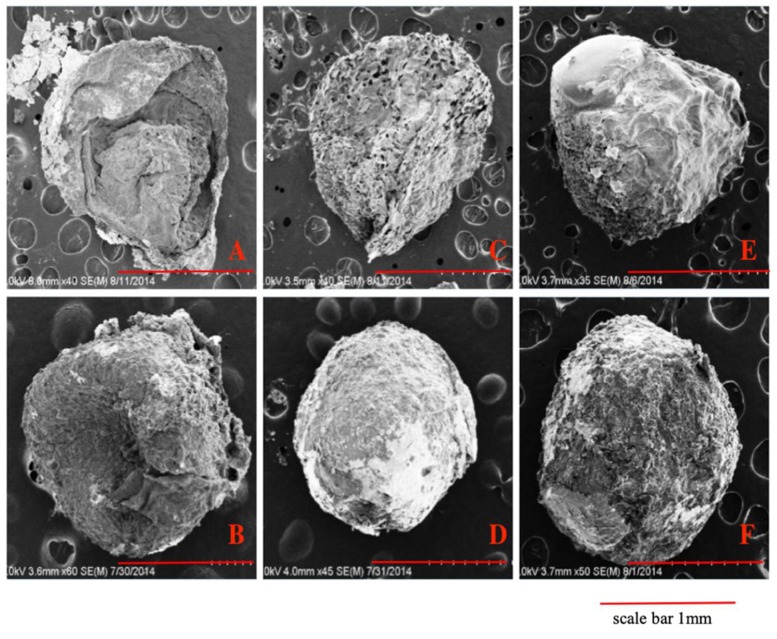
SEM images of pure chitosan hydrogel beads with increased chitosan concentration (**A**,**C**,**E**) and chitosan/ Halloysite nanotubes (HNTs) wt./wt. composites (**B**,**D**,**F**). (**A**) 3% chitosan (CS), (**B**) 3% CS/2% HNTs, (**C**) 4% CS, (**D**) 4% CS/2% HNTs, (**E**) 5% CS, (**F**) 5% CS/ 2% HNTs.

CS and CS/HNTs hydrogels were dropped into 10% tripolyphosphate (TPP) solution. The ionic cross-linking happened between the NH_3_^+^ site of chitosan and OH^−^ site on TPP. After 10 minutes cross-linking process, spherical hydrogel beads were formed with an average diameter of 3.38 ± 0.28 mm. For SEM analysis of surface and structural features of the hydrogel beads, beads were pretreated by lyophilization. Due to the pressure changes in the vacuum chamber during lyophilization, some hydrogel bead formulations collapsed and lost their spherical shape. A low concentration of chitosan (3% CS, Figure 1A) was barely able to preserve its original spherical shape as the hydrogel walls collapsed. However, as the chitosan concentration increased, the hydrogel beads structure provided some resistance to deformation and collapse of the hydrogel wall structure (Figure 1C,E). In contrast, the increased addition of HNTs may have enabled the CS/HNT hydrogels to resist deformation and preserve a more rounded microbead shape. HNTs may have interacted with chitosan to form stronger walls and provide more support to the hydrogel matrix. Also, the increased addition of HNTs produced a rougher surface (compare Figure 1D,F). 

### 2.2. Degradation

**Figure 2:**
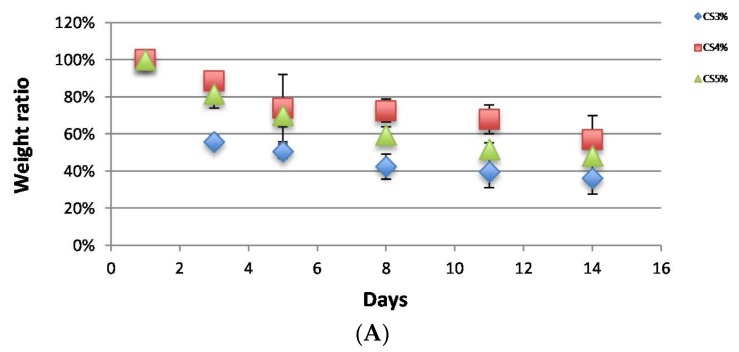

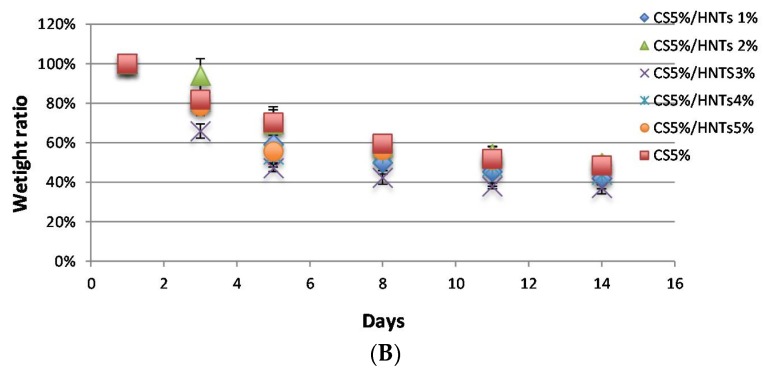
Biodegradability of CS and CS/HNTs in a lysozyme solution (1 mg/mL). (**A**). The weight ratio of hydrogel beads consisted of pure chitosan (3%–5%). (**B**). The weight ratio of hydrogel beads consisted of 5% CS with different ratios of HNTs (1%–5% wt./wt.).

CS and CS/HNTs hydrogel beads did not exhibit any weight loss when incubated in PBS without lysozyme (data not shown). When the hydrogel beads were incubated with lysozyme, they degraded gradually as expected, and their weight ratio decreased, as shown in Figure 2. Among the pure CS group (Figure 2A), 3% CS degraded fast after the first 3 days, and this speed was significantly faster than 4% and 5% CS. (one-way ANOVA, *p* = 2.37 × 10^−6^) However, after 14 days incubation, there was no significant difference in the final weight ratios (*p* = 0.09). These results show that the biodegradation ability of chitosan does not change with increases in CS concentration. Simultaneously, there was no significant difference between CS (5%) and CS/HNTs (5%/1%–5%, *wt*./*wt*.) (Figure 2B). The addition of HNTs did not affect CS biodegradability.

### 2.3. Tensile Property

**Figure 3:**
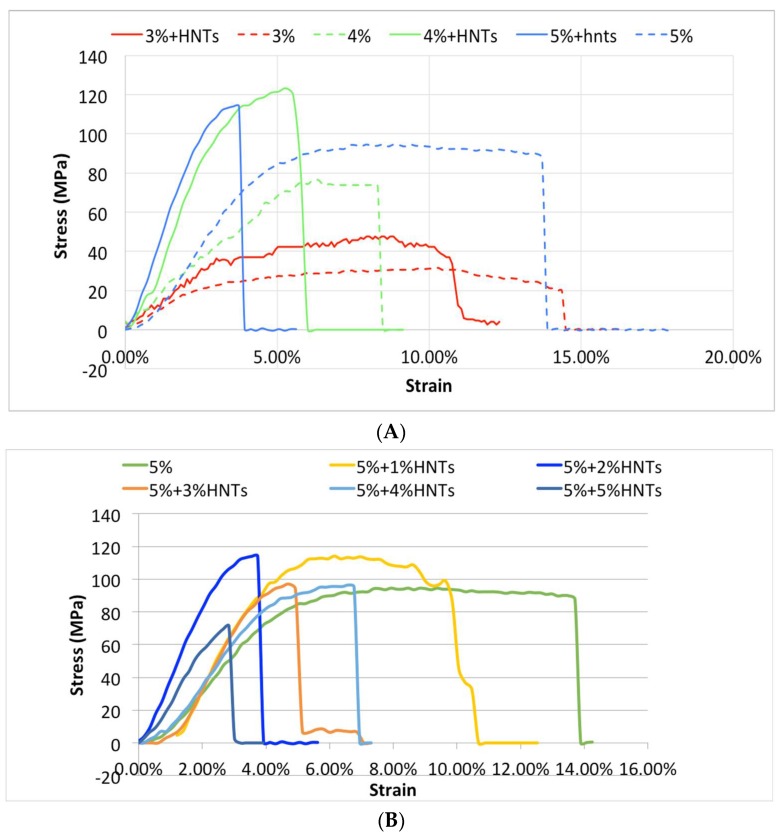
The stress-strain profile of CS and CS/HNTs. (**A**) Tensile test of pure chitosan (CS) with the HNT additive groups (CS/HNTs). In this graph, every CS/HNT compound has a higher stress value compare to CS group. (3% CS/2% HNTs > 3% CS, 4% CS/2% HNTs > 4% CS, 5% CS/2% HNTs > 5% CS). Simultaneously, a higher concentration of chitosan showed higher stress values (5% > 4% > 3%), however, the strain values displayed a different response (5% < 4% < 3%). (**B**) 5% CS/1% HNTs showed a major improvement in elongation, while 5% CS/2% HNTs showed the greatest improvement in strength. Increasing of number of HNTs gradually decreased its reinforcement ability, until at these concentrations (CS/HNTs (5% CS/ 5% HNTs), the nanocomposites was weaker and more fragile than pure CS. The step-wise failure behavior (slippage) at the end of each profile represents the fracture point of each specimen.

CS and CS/HNTs hydrogels (10 mm × 20 mm × 0.02 mm) were subjected to uniaxial testing using a CellScale Univert™ material testing device at a speed of 10 mm/min (Figure 3A,B). As expected, higher chitosan concentrations imparted higher tensile stress resistance (σ), which is represented as MPa, while a lower concentration provided higher elongation (ε) represented as strain (%) in Figure 3A. The addition of HNTs (2% *w*/*v*) enabled higher force loading but reduced elongation (Figure 3A). When 5% chitosan was mixed with HNTs at different ratios (from 1% to 5% *w*/*v*), a lower concentration of HNTs increased the nanocomposites tensile strength and elongation, while this reinforcement decreased with increasing HNT addition. When the HNTs were increased to 5%, the CS/HNTs nanocomposite showed even weaker resistance than pure chitosan (Figure 3B). 

**Figure 4:**
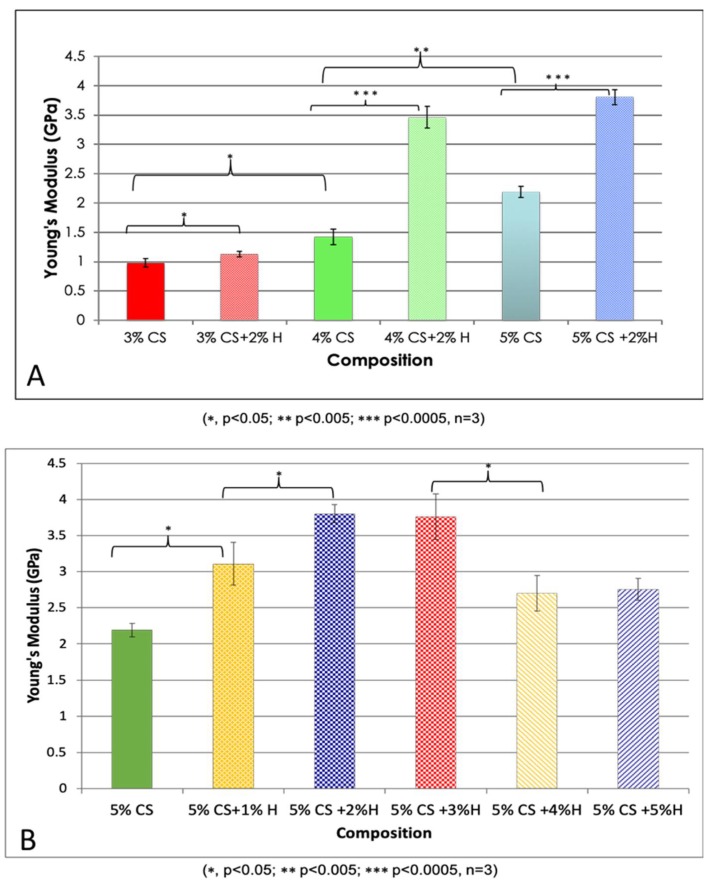
Young’s Modulus values of CS and CS/HNTs. (**A**) Different concentration of chitosan (3%–5%) and their combination with 2% HNTs wt./wt. (**B**) 5% CS combine with HNTs at different ratio (1%–5%). The changes are similar to the stress-strain profile. Young’s modulus value increased with HNTs increasing at low concentration (1%, 2%), as HNTs over 3% in composition Young’s modulus value decreased significantly.

The Young’s modulus values were calculated according to the stress (σ) and strain (ε) values: Young’s modulus = stress/strain. Based on three repetitive measurements, the average values and standard deviation of Young’s modulus were calculated, and the differences were compared and are presented in Figure 4. Using a one-way ANOVA analysis, there was a significant difference in Young’s modulus among the different chitosan concentrations (*p* = 0.00002) supporting the conclusion that chitosan concentration is a crucial factor affecting Young’s modulus. In addition, HNT addition significantly improved the tensile strength of CS, (*p* = 0.038, 3% CS vs. 3% CS/2% HNTs; *p* = 0.001, 4% CS vs. 4% CS/2% HNTs; *p* = 0.00006, 5% CS vs. 5% CS/2% HNTs). However, increases in tensile resistance after HNT addition gradually decreased as the concentration of HNTs increased to 5%, there was a weakening in hydrogel material properties.

### 2.4. Swelling Ratio

**Figure 5:**
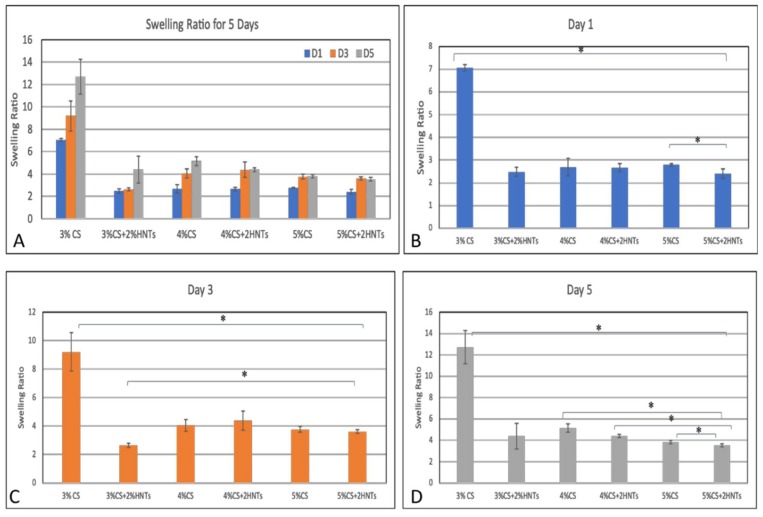
Swelling ratio for each hydrogel. The overall changes are presented in summary figure (**A**). The swelling ratio for Day 1, Day 3 and Day 5 (**B**–**D**, respectively). The symbol * indicates a significant difference (*p* < 0.05, *n* = 3). Error bar represents standard deviation.

Swelling behavior is a consequence of the interaction between a hydrogel and water. The rate of swelling is determined by several physicochemical parameters which include hydrogel porosity, the mature of its porous structure, interactions between its polymer chains and the water molecules. A higher swelling ratio indicates more free volume exists in the hydrogel, and the free volume between knots are affected by the crosslink density. Thus, swelling ratio is also used to measure crosslink density. In this study, swelling ratio was calculated using the fractional increase in the weight of the hydrogel. In Figure 5, after 1, 3, and 5 days incubation, the swelling ratio of low chitosan concentration (3%CS) is significantly higher than the hydrogel that is composed of high chitosan concentration (3% CS> 4% CS> 5%CS). The addition of HNTs significantly reduced hydrogel swelling ratio (3%CS >3%CS + 2%HNTs, 4%CS > 4%CS + 2%HNTs, 5%CS >5%CS+2%HNTs). Thus, lower concentration of chitosan had less crosslink density, and the addition of HNTs increased the crosslink density. Furthermore, the swelling ratio change indicates that the porosity and porous structure were also affected by chitosan concentration. 

### 2.5. Drug Release

Gentamicin was selected as a model for drug release. The final drug loading efficiency of gentamicin loaded into HNTs was 13.96 ± 1.1%. The pattern of gentamicin release from the chitosan/HNT beads was used to validate what composition would serve optimally as a drug delivery system. As the results show in Figure 5, gentamicin released from HNTs had a burst release in the first 10 h, while CS/HNTs provided a more stable and extended drug release profile. According to one-way ANOVA analysis, there was no significant difference in drug release capability among 3% CS, 4% CS, and 5% CS at first 56 h, but at 104 h, there was a significant difference between them (*p* = 0.018), which indicates a higher concentration of chitosan could provide a longer drug release time.

### 2.6. Bacterial Growth Inhibition Testing 

**Figure 6:**
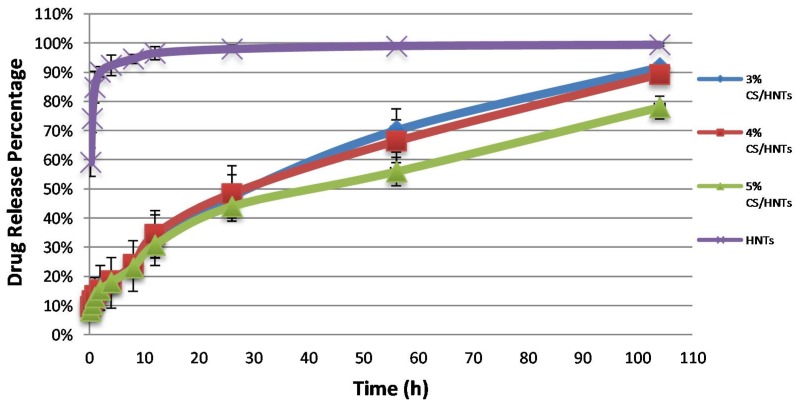
Accumulated drug release profiles for CS/HNTs hydrogels and pure drug-loaded HNTs. Every group contained the same amount of drug-loaded HNTs.

**Figure 7:**
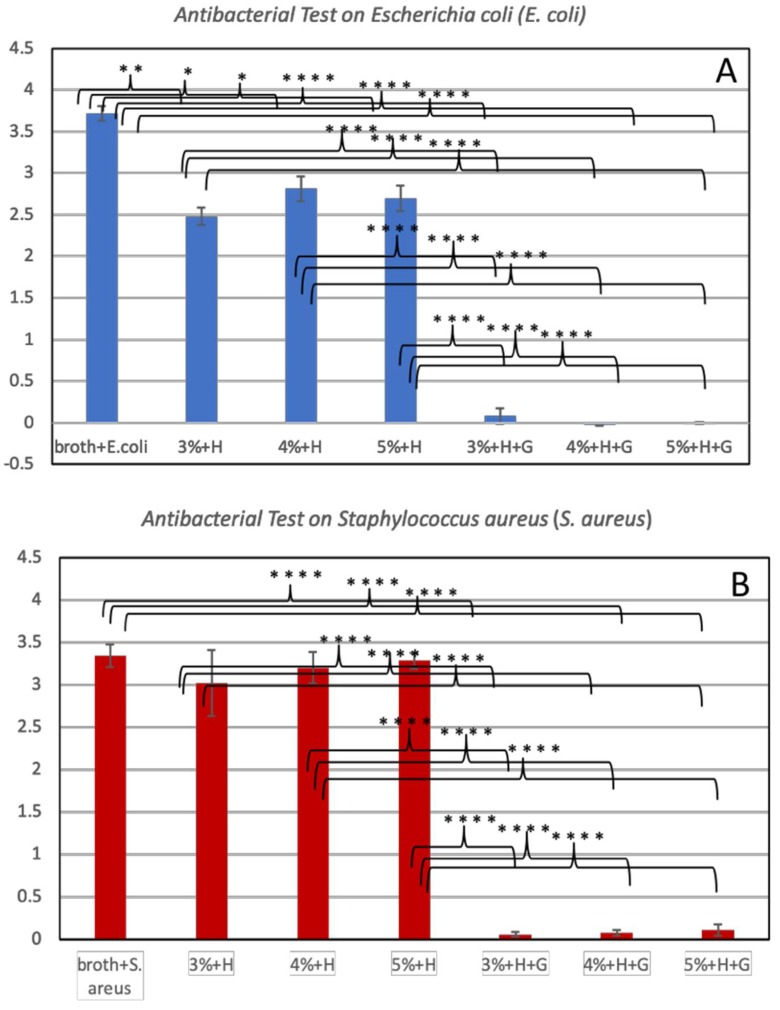
Antibacterial test on *E.coli* (**A**) and *S. aureus* (**B**). The absorbance values at 630 nm for: pure bacteria suspension (broth + *E. coli,* broth + *S. aureus*); CS/HNTs without antibodies (3%+H, 4%+H, 5%+H), CS/HNTs with antibodies (3%+H+G, 4%+H+G, 5%+H+G). Error bar with standard deviation. (∗ *p* < 0.05; ∗∗ *p* < 0.005; ∗∗∗∗ *p* < 0.00005, *n* = 3).

The CS/HNT hydrogels ability to inhibit the growth of the gram-positive bacteria (*S. aureus*) and gram-negative bacteria (*E. coli*) were studied. The optical density (OD) values at 630 nm of each group were recorded and are presented in Figure 6, with a higher absorption value indicating a higher concentration of bacteria. The pure bacteria suspensions, *E. coli* and *S. aureus*, without any treatment showed continued growth over a 24-h period and served as the controls. CS/HNTs hydrogels, with or without the antibiotic (gentamicin), were compared with the controls. Our results indicate that CS/HNTs hydrogels without gentamicin inhibited the growth of *E.coli* (Figure 7A). However, inhibition of bacterial growth was considerably less when tested against *S. aureus* (Figure 7B). In contrast, gentamicin-loaded CS/HNTs hydrogels showed significant antibacterial growth resistance against both bacterial species over an extended time period. 

### 2.7. Live/Dead Assay

**Figure 8:**
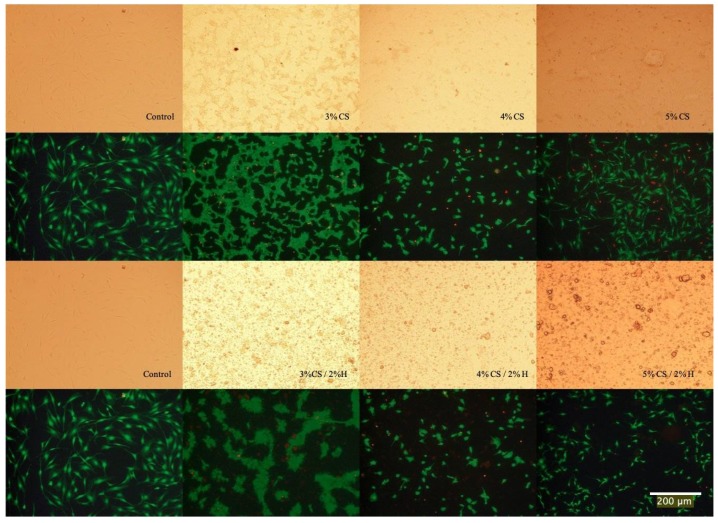
Live/Dead Assay of cells cultured on CS and CS/HNTs. Compared to control, there are live cells (green) observed on the film-coated plates, but the number of dead cells (red) has increased as compared with the control plate but remain few in number. This indicates cells can adhere and proliferate on CS and CS/HNTs substrate. However, cellular morphology was influenced by the surface features and physicochemical properties of substrate.

**Figure 9:**
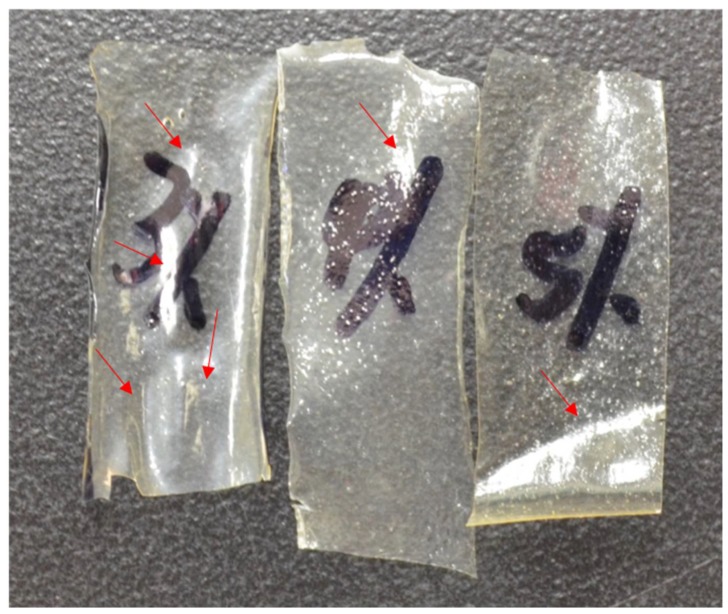
Picture for chitosan films after they were crosslinked and dried. The red arrows point to the wrinkles on the film.

The Live/Dead assay was applied to pre-osteoblast cultures as a means for estimating the cell viability after exposure to the chitosan and chitosan/HNT composite films. Cell cultures were then photodocumented and the fluorescently-labeled cells also provided an excellent opportunity to observe and record cell adhesion and spreading. As the images in Figure 7 show, when compared to control culture wells, cells cultured on CS/HNTs substrates showed excellent cytocompatibility with little cytotoxic effect. There are no major differences in observed cellular behavior among control and hydrogel groups with the exception of the 3% CS and 3% CS/ 2%H groups. Cells cultured on these films appeared to cluster and form small colonies (Figure 8). This behavior may due to surface features or physicochemical properties of the films. As is shown in Figure 9, among three different concentrations, 3% chitosan has more wrinkles. This observation is consistent with what we found above: a lower concentration of chitosan had weaker mechanical properties. When cell culture plates were coated with different concentrations of chitosan, lower concentrations of chitosan also presented a reduced degree of stiffness as observed during manual handling of these films. Furthermore, it was more difficult for the softer material to maintain its scaffold structure. During the crosslinking process, multiple micro-scale wrinkles were formed in 3% CS and 3% CS / 2% HNT hydrogels. The substrate surface features and physicochemical properties may have cellular behaviors resulting in the observed cell clusters (Figure 7).

## 3. Discussion 

The goal of this study was to fabricate a nanoclay-enhanced hydrogel for potential use as a biodegradable drug delivery system. Critical in our design was to produce hydrogel films with suitable strength enabling a range of application such as topical application or injection. Clay nanoparticles are present in nature in several different morphologies depending on the nature of their layered structure. Clay nanoparticles are being actively researched for their potential in a variety of biomedical applications, in particular, drug delivery. The most well-known of these nanoparticles include kaolinite, montmorillonite, and halloysite [43,44]. Kaolinite is an abundant and inexpensive clay mineral and has a long history in drug delivery applications [45]. Kaolinite has been used in many pharmaceutical applications either as an excipient or an active ingredient because of its excellent physical, chemical, and surface physicochemical properties [44,45]. Its application within composite drug delivery systems include antimicrobial [46], anticancer [47], skeletomuscular and geriatric diseases [45] as well as a bioactive agent for the treatment of some common diseases. Kaolinite and chitosan nanocomposites have seen significant research activity [48,49]. 

Montmorillonite clay (MMT) belongs to the smectite group with tetrahedral silica sheets layered between alumina octahedral sheets at a ratio of 2:1, respectively [50]. It has a large specific surface area, exhibits good absorbance ability, high cation exchange capacity, adhesiveness, and drug-carrying capability [49,50]. Drug incorporation into MMT by adsorption into its interlayer-spaced structure within by replacement of the water molecules, and also on the surface. The most important interactions taking place between the two components of the hybrid system are ionic [50,51]. Chitosan MTT composites have developed as drug delivery systems for antimicrobial [52], cancer [53], gastrointestinal [53], osteoarthritis [54], and wound healing applications [55]. Emerging in 2012 as a potent nanocarrier and nanocontainer, halloysite is tubular aluminosilicate nanoparticle and has been under intense study as an agent for the sustained release of antibiotics, chemicals, chemotherapeutic agents, and growth factors [50,51]. HNTs typically display an inner diameter ranging from 15 to 50 nm, an outer diameter ranging from 30 to 50 nm, and lengths between 100 and 2000 nm [56,57,58]. HNTs have been shown to serve as a nanocontainer with vacuum-trapped drugs, bioactive agents, and other substances, and these are released in a sustained manner [59,60,61,62]. 

In this study, the effects of chitosan and HNTs concentration and combination ratios of these materials on the mechanical properties of a hydrogel composite and its drug release capability were analyzed. Our results suggest that a higher chitosan concentration produced a more uniform bead shape and drug release capability. Other studies have shown that ionic gelation [63,64] (Al^3+^, Ca^2+^, and Zn^2+^) or chemical cross-linking [65,66] can also produce strong beads with a more spherical shape. Lower concentrations of chitosan and HNTs produce beads that were very soft and irregular in shape.

**Figure 10:**
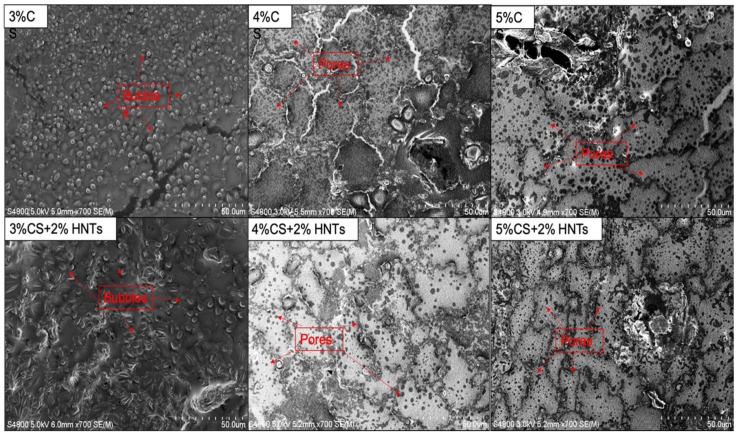
SEM images of cross-section for different hydrogel composites. The arrows in the picture of 3% chitosan (3%C) and its HNTs composites (3%CS + 2%HNTs) point to the example of bubbles. The arrows in the picture of 4% chitosan (4%C) and 5% chitosan (5%C) and their hydrogel composites (4%CS + 2%HNTs and 5%CS + 2%HNTs) point to the example of pores.

Higher chitosan concentration also created a hydrogel with smaller pore sizes. Hydrogels with smaller pores were also less deformable than gels with larger sized pores. A similar finding was reported by Chiu et al., (2013) with poly (ethylene glycol)-co-(l-lactic acid) hydrogels [67]. To verify this potential explanation, we took the cross-section SEM images for the hydrogel beads. In Figure 10, both 4% and 5% chitosan and their HNTs composite hydrogels have a lot of small pores. There is no significant different in pore size between 4% and 5% chitosan and its HNTs composites. However, 3% chitosan and its HNTs composite hydrogels have numerous bubbles instead of pores, and the bubbles are much bigger than the pores formed in 4% and 5% chitosan hydrogels. Those bubbles were formed during the drying process. If all the bubbles break, big size of pores would remain. This observation partly supports our hypothesis. 

As expected, the deformability also increased with polymer content, which agrees with the literature showing increasing crosslink density with increased polymer content [68,69]. The mechanical properties also diminished rapidly during incubation, suggesting a bulk mechanism of degradation, which is consistent with our swelling (Figure 5) and pore size observations (Figure 10). The addition of HNTs to chitosan did not affect pore structure or porosity of the scaffolds, a result also reported by Liu et al., (2012) [17]. 

The objective of the degradation study was to determine whether the HNTs addition inhibited or increased chitosan degradation. Our research showed there was no significant effect with HNT addition on degradation, indicating the stability of the chitosan/HNT and the predictability of biodegradation rates dependent on the final composites. As anticipated, HNT addition did contribute improvement in chitosan hydrogel tensile properties. In two previous studies, HNT addition to the chitosan matrix also significantly enhanced compressive strength, compressive modulus, and thermal stability [42,66]. HNTs are widely used as a polymer bulk filler added to significantly improve the mechanical, swelling, water uptake, thermal, drug-loading efficiency of the composite matrices [17,24,32].

**Figure 11:**
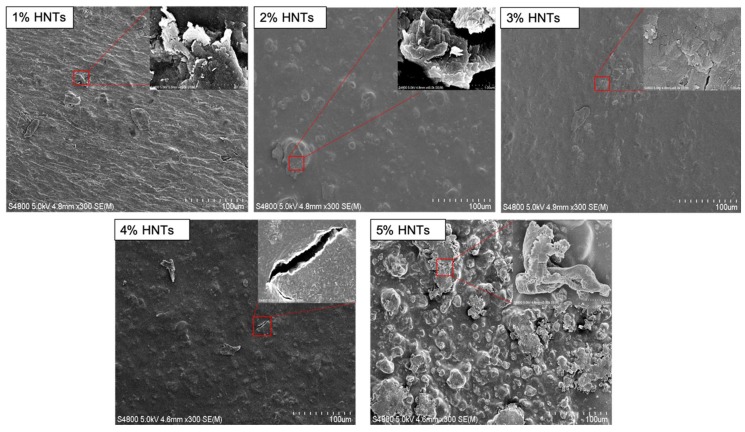
SEM images of hydrogel surface that consisted by 5% chitosan and combined with HNTs at different rations (1% to 5%). The pictures in the right corner are the zoom in pictures of the selected areas.

In this study, however, when HNT concentration exceeded an absolute value, hydrogel deformability decreased sharply. In this study, 2% wt./wt. combined ratio showed the best response to tensile testing. The results of degradation can also be explained along the same lines. The 2% wt./wt. HNTs-chitosan hydrogels also showed the slowest rate of degradation. Our observation of HNT response to deformability may be due to inadequate dispersion of HNTs in the chitosan matrix. [5] The interfacial binding between HNTs and chitosan is achieved by hydrogen bonding and electrostatic interactions [17]. A uniform dispersion results in a uniform interfacial-binding matrix, which is favorable to force conduction. In contrast, too many nanotubes inhibit the dispersion state and create interfacial gaps, which are easy to break. This phenomenon is clearly presented in Figure 11: 5% chitosan combined with HNTs at different rations (1% to 5%), HNTs clusters were observed by SEM. The hydrogel films with higher concentration HNTs have bigger and more HNTs clusters. Those clusters resist the force conduction and may result in gaps, which is represented in the insert picture of Figure 11-4% HNTs. Instead of reinforcing the biomaterials, exceeded addiction of HNTs weakening biomaterials original mechanical properties.

In terms of the chitosan/HNT composite’s potential as a drug delivery system, the results of the drug release profile analysis showed a doped drug could be released in a sustained fashion, and bacteria growth inhibition tests indicate that the release of gentamicin was able to inhibit bacterial growth. In summary, the chitosan-HNTs hybrid hydrogel exhibited better mechanical properties as compared to pure chitosan hydrogels, and their combination showed a more sustained ability in drug release. Chitosan and HNTs are eco-friendly and biocompatible materials, [12,13,19,22,23] and with increases in their mechanical properties, they will have increased use in clinic treatments. For instance, coating implants and providing a long-term drug delivery to prevent wound infection. Furthermore, instead of antibiotics HNTs could be loaded with growth factors designed to direct cell migration by chemotaxis or induce differentiation. 

## 4. Conclusions

Chitosan-based hydrogels are being used due to biodegradable properties, and ability to that mimic the extracellular matrix of many tissues. However, the use of chitosan hydrogels has been limited by their inherent mechanical weakness. In this study, the effects of increased chitosan and HNT concertation on selected mechanical properties of chitosan/HNT hydrogels, with and without gentamicin addition. HNTs are widely employed as a bulk filler to improve the performance characteristics of many polymers. HNTs have also been shown to be a viable nanocontainer able to provide sustained release of antibiotics, chemicals, and growth factors. The addition of HNTs to chitosan hydrogels improved the gels’ mechanical properties. Chitosan/HNT gentamicin-doped hydrogels enabled sustained drug release and were effective in reducing bacterial growth. Our doped clay/chitosan nanocomposite may overcome the limitations of traditional anti-bacterial hydrogels by providing a focal drug delivery and sustained release of drugs, singly or in concert, or a suite of drugs or drug/growth factor combinations. Definitive conclusions from our study must be made with a degree of caution as the sample numbers in our studies were limited.

## 5. Experimental Section

### 5.1. Drug Loading

For drug loading, gentamicin sulfate (GS, Sigma Aldrich, St. Louis, MO, USA) was vacuum-loaded into HNTs. HNTs (250 mg/mL.) were mixed with a 2 mL GS solution (250 mg/mL). The mixed suspension was placed in a vacuum and the suspension was vacuumed overnight. The gentamicin contained in the supernatant was measured to determine the drug loading efficiency.

Drug loading efficiency = Gentamicin in supernatant/Total amount of gentamicin.

### 5.2. HNTs-Chitosan Hydrogel Construct

Low molecular weight chitosan (Sigma Aldrich) was dissolved in 4% critic acid solution (Fisher Scientific, Houston, TX, USA) to form three chitosan concentrations: a 3%, 4% and 5% *w*/*v* solution. Different concentrations of chitosan were combined with HNTs, with the concentration of HNTs ranging from 1% to 5%. Hydrogels were formed by crosslinking the mixture solution with 10% tripolyphosphate (TPP) (Sigma Aldrich).

### 5.3. Scanning Electron Microscopy (SEM) Study 

The HNTs-chitosan mixture and pure chitosan solution (200 μL) were dropped into a 10% TPP solution to produce similar sized droplets. After 10 minutes, the beads had formed, they were then frozen at −20 °C for 24 h and then lyophilized. The structures of hydrogel beads were studied using SEM (AMRAY SEM, Model: 1830, SEMTech Solutions, North Billerica, MA, USA).

### 5.4. Degradation Analysis

0.25 mL hydrogels were cross-linked into micro-beads and incubated in PBS at 37 °C for 24 h first. Their initial weight were measured W_d1_ after beads air-dried for 30 minutes on filter paper. Then, the hydrogels were divided in two groups, one group was incubated in PBS, another group was incubated in 1 mg/mL lysozyme/PBS solution at 37 °C. Their weights were measured every 2 to 3 days and recorded as W_dx_. This study was continued for 14 days. The remained weight ratio for each sample was calculated as:

Weight ratio = W_dx_/W_d1_.

### 5.5. Tensile Properties

The chitosan-HNTs mixture and pure chitosan solution were poured into the same size mold, after they had totally dried, a 10% TPP solution was added for cross-linking chitosan. The crosslinked hydrogels washed with DI water for 3 times, then put on filet paper for air-drying. The prepared films were cut into similar sizes (10 mm × 20 mm), and the average thickness was 0.02 mm. The tensile strength (**σ**) and elongation (**ε**) of hydrogels was measured by CellScale Unit with 200 N load cell at a speed of 10mm/min. Young’s modulus (***E***) were calculated based on the equation of ***E*** = **σ**/**ε**. At lease 3 tests for each composite. 

### 5.6. Drug Release Study

10 mg of drug-loaded HNTs were mixed with 0.5 mL chitosan solution and cross-linked with 10% TPP solution for 30 minutes. After rinsed by DI water for 3 times, all the samples were incubated in 2 mL PBS at 37 °C. When collecting drug release aliquots of each solution was removed and filled with fresh PBS. Gentamicin containing samples combined with o-ophthalaldehyde (OPTA) solution and 50% isopropyl at a ratio of 1:1:1 by volume, then measured at 340 nm wavelength.

### 5.7. Swelling Ratio

Hydrogel beads composed of pure chitosan and chitosan/HNTs composites were prepared as above. Each hydrogel bead was incubated in 200ul phosphate buffer saline (PBS) at 37 °C for 5 days. At day 1, 3, and 5, the swelling ratio of chitosan and chitosan/HNTs hydrogel composites were then determined by the following equations. Where, *W_s_* represents the weight of swollen hydrogel after incubation in PBS, and *W_d_* represents the weight of dried hydrogel after swelling.

Swelling ratio = (Ws − Wd)/Wd

### 5.8. Bacterial Inhibition Growth Test

Cross-linked hydrogel beads, consisting of CS/HNTs and drug-loaded CS/HNTs, were placed in 1 mL *Escherichia coli (E. coli)* and *Staphylococcus aureus (S. aureus)* suspension and incubated with nutrient broth (NB) and Mueller Hinton broth respectively at 37 °C for 24 h. Pure bacteria suspension without any treatment set as control, pure broth set as blank. The optical density (OD) of samples were measured at wavelength of 630 nm at time point of 0, 3, 16, and 24 h. Each sample has three replicates.

### 5.9. Live/Dead Cytotoxicity Assay 

48 well plates were pre-coated with CS or CS/HNTs hydrogel films, then MC3T3 cells (ATCC) were seeded at a density of 1 × 10^5^/mL. Culture wells without any film coating were used as controls. All cultures were then incubated at 37 °C with 5% CO_2_ for 24 h. The Live/Dead assay (Life Technologies, Carlsbad, CA, USA) was applied according to manufacturer’s directions to assess any potential for cytotoxicity. 

### 5.10. Statistical Analysis

Statistical analysis was conducted by using one-way ANOVA or Student’s *t*-test. All of the quantitative experiments were performed in triplicate or repeated three times. Data were expressed as mean Å+/− the standard error. Significance between experimental groups and/or controls was determined by one-way analysis of variance. A *p*-value less than 0.05 was considered statistically significant.
